# Nucleosome Presence at AML-1 Binding Sites Inversely Correlates with Ly49 Expression: Revelations from an Informatics Analysis of Nucleosomes and Immune Cell Transcription Factors

**DOI:** 10.1371/journal.pcbi.1004894

**Published:** 2016-04-28

**Authors:** Andrew Wight, Doo Yang, Ilya Ioshikhes, Andrew P. Makrigiannis

**Affiliations:** 1 Department of Biochemistry, Microbiology and Immunology, University of Ottawa, Ottawa, Ontario, Canada; 2 Institute of Systems Biology, University of Ottawa, Ottawa, Ontario, Canada; 3 Department of Microbiology and Immunology, Dalhousie University, Halifax, Nova Scotia, Canada; Rutgers University, UNITED STATES

## Abstract

Beyond its role in genomic organization and compaction, the nucleosome is believed to participate in the regulation of gene transcription. Here, we report a computational method to evaluate the nucleosome sensitivity for a transcription factor over a given stretch of the genome. Sensitive factors are predicted to be those with binding sites preferentially contained within nucleosome boundaries and lacking 10 bp periodicity. Based on these criteria, the Acute Myeloid Leukemia-1a (AML-1a) transcription factor, a regulator of immune gene expression, was identified as potentially sensitive to nucleosomal regulation within the mouse Ly49 gene family. This result was confirmed in RMA, a cell line with natural expression of Ly49, using MNase-Seq to generate a nucleosome map of chromosome 6, where the Ly49 gene family is located. Analysis of this map revealed a specific depletion of nucleosomes at AML-1a binding sites in the expressed Ly49A when compared to the other, silent Ly49 genes. Our data suggest that nucleosome-based regulation contributes to the expression of Ly49 genes, and we propose that this method of predicting nucleosome sensitivity could aid in dissecting the regulatory role of nucleosomes in general.

## Author Summary

The nucleosome—a large protein complex with DNA wound around it—is the fundamental unit of genomic organization in the eukaryotic cell. More than just a DNA organizer, however, nucleosomes may control gene expression by interfering with the cell’s ability to access the wound-up DNA, as shown by recent research. In this report, we demonstrate a computational method for predicting which elements of the genome are sensitive to regulation by nucleosomes. As a proof-of-concept, we identify AML-1a binding sites—important sequences in DNA regulation—as being specifically nucleosome sensitive. We then show that AML-1a sites are specifically depleted of nucleosomes when a gene is expressed, indicating the ability for nucleosomes to suppress the expression of that gene. This finding confirms that nucleosomes are likely involved in genome regulation, and provides a method for predicting which areas of the genome are probably affected most by nucleosomes. This paper also highlights the usefulness of the Ly49 gene family in testing computer-derived genomic predictions, and is of interest to anyone studying how gene expression is regulated from cell to cell.

## Introduction

The nucleosome, comprising an octameric protein core surrounded by ~147 bp of DNA, is the fundamental unit of genomic organization in the eukaryotic cell [1,2]. This organizational paradigm lays the groundwork for higher-order chromatin compacting, ultimately allowing the dozens of centimetres of DNA in a typical mammalian cell to be packaged into a micrometer-sized nucleus [3]. Beyond this impressive organizational role, however, the nucleosome—a bulky and ubiquitous protein structure tightly bound to DNA [4]—is believed to occupy a central role in the regulation of gene transcription [5,6]. The histone core of the nucleosome contains some of the most heavily targeted proteins for post-translational modification. These modifications, or histone marks, encode the epigenome: a highly plastic, inheritable set of expression instructions that are responsible for the incredible diversity of cellular morphology and function that can arise from a single genome [7–9].

The role of histone modifications in regulating gene expression has received considerable attention since the discovery of the epigenome, and considerably more attention is still required to understand this complex regulatory network. Alongside these histone modifications, however, nucleosomes are also believed to regulate gene expression simply by their steric effects on the accessibility of a given sequence of DNA [10]. Originally, nucleosomes were believed to be evenly spaced every ~200 bp of DNA, emphasising the structural role they play [11]. Now, however, it is known that, while many nucleosomes do obey this simple spacing model, many others are specifically positioned within the genome, either by the action of chromatin remodeling enzymes [12] or by the basal affinity of a given stretch of DNA for a nucleosome [13–15]. The identity and target sequences of all chromatin remodeling enzymes are not yet known [16]; however, work from our lab and others have identified DNA sequence patterns that allow the modeling of basal nucleosome affinity for a given genetic sequence, allowing for *in silico* mapping of the ‘default’ nucleosome landscape of the genome [11,17]—that is, the nucleosome landscape before it is changed by remodelling enzymes.

In more specific terms, nucleosomes are understood to be positioned either statistically or specifically [18]. Statistical positioning means the nucleosome position is limited only by the adjacent nucleosome; if there is no adjacent nucleosome, the statistically positioned nucleosome could reside on any sequence of DNA. Conversely, specific nucleosome positioning means the nucleosome position is closely regulated by the underlying DNA sequences [11,13,14,19] or by nucleosome remodeling factors [12,20,21]. Various eukaryotic nucleosome positioning sequence (NPS) patterns have been proposed. These NPS are characterized by repeating nucleotide sequences in yeast [11,14,22], fly [23], and *C*. *elegans* [24]. Specifically, the periodic appearance of dinucleotides, such as AA or TT, in every 10 bp is a commonly observed sequence pattern that allows the tight bending of DNA around the histone octamer core [25–27]. Many statistical methods incorporating various parameters, such as Support Vector Machine [13], DNA deformation energy [8], a thermodynamic model including interactions between adjacent nucleosomes [28], or the hidden Markov Model [29], were used to propose the NPS patterns which predict nucleosome positions from the underlying genomic sequences.

Our goal has been to study how changes to this ‘default’ sequence-determined nucleosome landscape correlate with gene expression. To that end, we have investigated the effects of nucleosome positioning on the expression of a family of immune genes, the Ly49 receptors. Ly49 receptors—and their human analogues, the killer-cell immunoglobulin-like receptors (KIR)—are expressed on natural killer (NK) and other immune cells. These receptors interact with the class-I major histocompatibility complex (MHC-I). This sensitivity to MHC-I expression levels allows NK cells to distinguish healthy cells from cancer or virus-infected cells, and is vital to the innate immunosurveillance performed by NK cells [30,31].

Aside from its importance in immunology, the Ly49 family has several traits that make it an ideal model for our investigation of transcriptional regulation by nucleosomes. The Ly49 genes all reside in a 500,000 bp region of chromosome 6, and have very similar transcription factor requirements. However, expression of an individual Ly49 gene is stochastic, such that each NK cell acquires a unique repertoire of Ly49 receptors during development and then maintains this repertoire throughout its life [32–34]. This therefore presents a model system in which nucleosome differences in expressed genes can be compared to their silent neighbours, which require the same transcription factors and would be equally impacted by large-scale events such as chromosome looping, the actions of locus-control regions, and any technical errors. Additionally, a common NK-T cell line—called RMA—naturally expresses only Ly49A and none of the other Ly49, providing a convenient model for this line of study. While interesting, the stochastic expression of Ly49 in primary NK cells provides a number of technical challenges for an analysis such as this, favouring RMA as our model for this study.

In mice, this stochastic expression is achieved in part by the coordinated activity of two or three promoters for each gene [35]. Immature NK cells use the first promoter (Pro1), which is a bidirectional promoter consisting of a conserved transcription factor binding platform: a 5’ and a 3’ TATA box each flanked by C/EBP binding sites, separated by a central AML-1 and NFκB binding site [35]. The forward and the reverse directions for Pro1 compete for the same transcription complex, which by chance may assemble and transcribe in only one of the two directions. Forward transcription is believed to dislodge an inhibitory complex around the downstream second and/or third promoter [35,36], which then drives expression of that Ly49 for the rest of the NK cell’s life. Conversely, reverse transcription means the inhibitory complex is present at a key developmental moment, forever barring the NK cell from expressing that Ly49 [35]. The relative strength of the forward and reverse promoters is believed to be modulated by the C/EBP binding sites. In highly expressed Ly49 receptors, their Pro1 has a ‘forward’ sequence with higher affinity for these transcription factors than their ‘reverse’ sequence, while receptors with low expression rates have a high-affinity ‘reverse’ and low-affinity ‘forward’.

While the above model nicely describes the observed Ly49 expression patterns on NK cells, a recent report has shown that Pro1 affinity does not always correlate with that Ly49’s expression level [37]. This finding suggests that some other, as-yet unknown factor may be at play in regulating Ly49 expression, giving rise to our interest in the effects of nucleosome positioning on Ly49 expression. While the transcription factor requirements for Ly49 expression have been described, we propose that certain necessary transcription factor binding sites within the Ly49 promoters or the enhancer are sensitive to the steric effects of nucleosome coverage. We expect that these sensitive binding sites will be preferentially enriched within predicted nucleosome-bound regions of DNA. Additionally, we and others have previously shown that some transcription factors are arranged ‘in-phase’ with nucleosomes, and so possess a noticeable pattern of 10 bp periodicity in nucleosome covered regions [38]. Since 10 bp corresponds to one turn of a DNA double helix [39], factors with this periodic pattern would be able to orient themselves to face ‘outward’ from the nucleosome even when covered and therefore be available for target protein binding [38]. Indeed, some TATA box binding sites have been shown to require this 10 bp phasing; disrupting the phasing of the binding site relative to local nucleosomes was shown to severely impact promoter function [40].

In this report, we identify AML-1a as a transcription factor with binding sites displaying both this preferential nucleosome coverage and lack of 10 bp periodicity. We then confirm, in a cell line that naturally expresses Ly49, that AML-1a sites are preferentially depleted of nucleosomes throughout the promoter/enhancer regions of expressed Ly49 genes when compared to the unexpressed genes within the same population, implicating nucleosome positioning as a possible mechanism to affect Ly49 expression *in vivo*.

## Results

### Patterns of nucleosome binding indicate possible regulation of Ly49 gene expression

Based on sequences generated previously in our lab, we predicted the basal nucleosome affinity of the C57BL/6 mouse Ly49 gene family. Using a hidden Markov model of nucleosome occupancy, we determined the probable nucleosome map for these regions (S1A Fig). These predictions show only a slight bias toward GC-rich regions (S1B Fig), and when tested against a published MNase-Seq dataset showing nucleosome occupancy in mouse hepatocytes, gave an overall accuracy of 48.8% (S1C Fig). That the accuracy of this prediction is so low is not surprising—the prediction only accounts for the genomic affinity for nucleosome binding, and cannot account for the action of chromatin remodelling factors.

**Fig 1:**
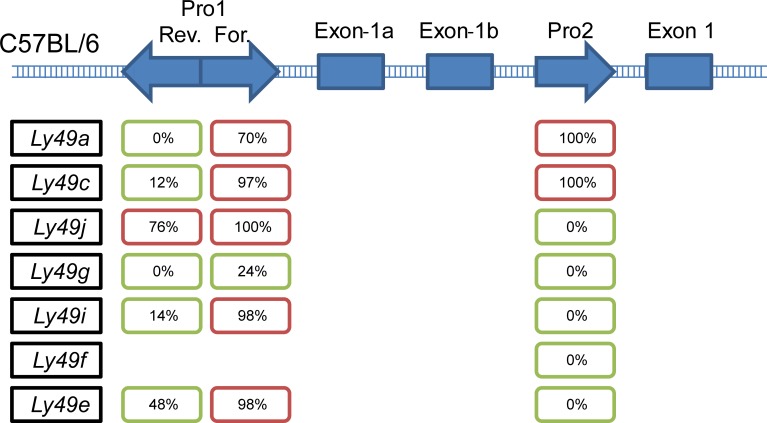
Predicted nucleosome hindrance at Ly49 forward promoter 1. Probable nucleosome position across the Ly49 gene family in the C57BL/6 mouse was determined using a 4^th^ order Hidden Markov model, based on the genomic sequence affinity for nucleosome binding. For each Ly49 gene with an annotated Pro1 and/or Pro2 region, the proportion of bases covered by a nucleosome is indicated. Regions with over half the bases covered are coloured red, while regions with over half the bases exposed are coloured green.

Having generated this sequence-based map of the default genomic nucleosome affinity, we next asked whether nucleosome positions, and their interaction with transcription factor binding sites, might contribute to Ly49 gene transcriptional regulation. For each of the Ly49 genes in the C57BL/6 genome with promoter annotations, we determined whether a pattern emerged when examining whether the promoter was predominantly nucleosome-bound or nucleosome-free (Fig 1). We also performed this analysis for Ly49 families from Balb/c, 129Sv, and NOD mouse strains, with similar results (S2 Fig). In most cases, the sequence at Pro1 reverse favours a free configuration, while Pro1 forward favours a bound configuration. We also noticed an AML-1a binding site located between the two promoters in virtually every case, agreeing with previous results for the B6 Ly49G promoter region [35].

### Identification of transcription factor binding sites with a predicted preference for nucleosome occupancy

**Fig 2:**
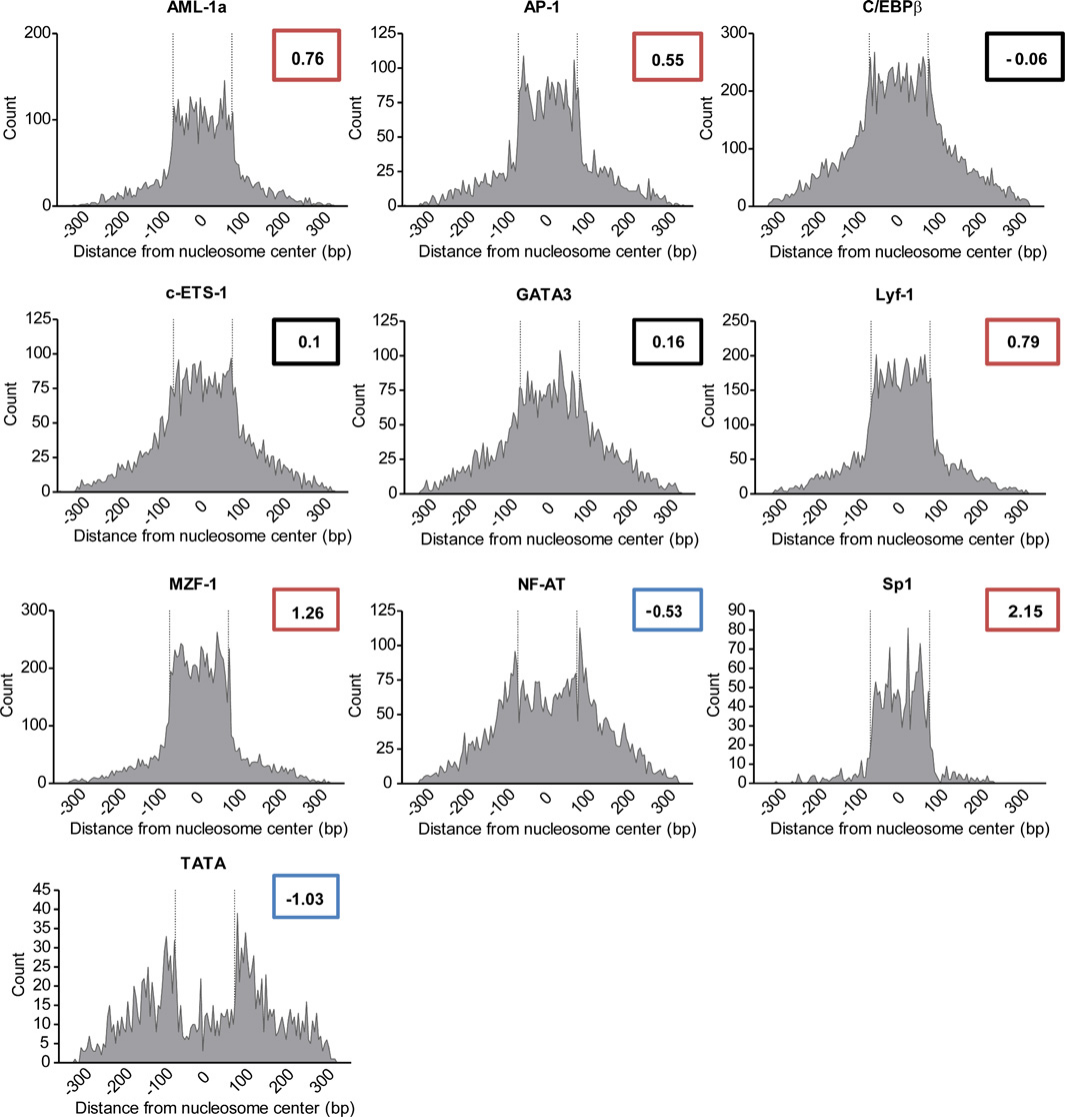
Proximity of transcription factor binding sites to predicted nucleosome centers within the Ly49 gene family. The C57BL/6 nucleosome map as generated in Fig 1 was compared individually to 17 transcription factors drawn from the TRANSFAC or JASPAR databases, plus TATA as a control. For factors with over 300 putative binding sites in the Ly49 gene family, a histogram is shown displaying each factor binding site’s distance to the nearest nucleosome. Dashed vertical lines indicate the nucleosome boundary. The kurtosis of each histogram is indicated. Histograms with a kurtosis less than -0.5 (blue) are considered to preferentially avoid nucleosome centers; those with kurtosis over 0.5 (red) are preferentially clustered in the center; and those between -0.5 and 0.5 (black) display no preference for nucleosome co-occupancy.

We next sought to determine whether certain TF binding sites within the Ly49 gene cluster are particularly sensitive to nucleosome coverage. First, since Ly49 receptors are thus far known only to be expressed in immune cells, and predominantly in natural killer lymphocytes, we selected 17 transcription factors with well-known functions in regulating lymphocyte gene expression (S3 Fig). Factors with known Ly49 interactions—including AML-1 and C/EBPβ [35]—were included, but to avoid bias, other common lymphocyte transcription factors were included. We also included TATA as a control, which is expected to be positioned away from nucleosome centers. For the whole Ly49 region, we plotted the distance from the center of each of these TF binding sites to the center of the predicted nucleosome binding site. Of the 18 factors, 10 gave sufficient signal for the Ly49 cluster to generate histograms plotting the spatial relationship between TF and nucleosome binding sites (Figs 2 and S4A). We observed three distinct patterns of TF-nucleosome spacing, based both on visual assessment and the calculated kurtosis—the mathematical measurement of a curve’s pointedness—of the histograms. First, some factors—namely, NF-AT and TATA—show a bimodal distribution and a negative kurtosis (< -0.5, indicating a flat curve) about the nucleosome center, with peaks existing at or near the nucleosome boundary. This suggests that these factor binding sites preferentially avoid nucleosomes, and are unlikely to be sensitive to nucleosome interference. Second, AML-1a, AP-1, Lyf-1, Sp-1, and MZF-1 show a tightened, monomodal distribution and positive kurtosis (> 0.5, indicating a pointed curve), with most factor binding sites being preferentially covered by the nucleosome. The rest of the factors analyzed were found to be normally distributed about the nucleosome dyad (kurtosis between -0.5 and 0.5), and so show no preference for nucleosome coverage.

### Identification of transcription factor binding sites displaying 10 bp periodicity

**Fig 3:**
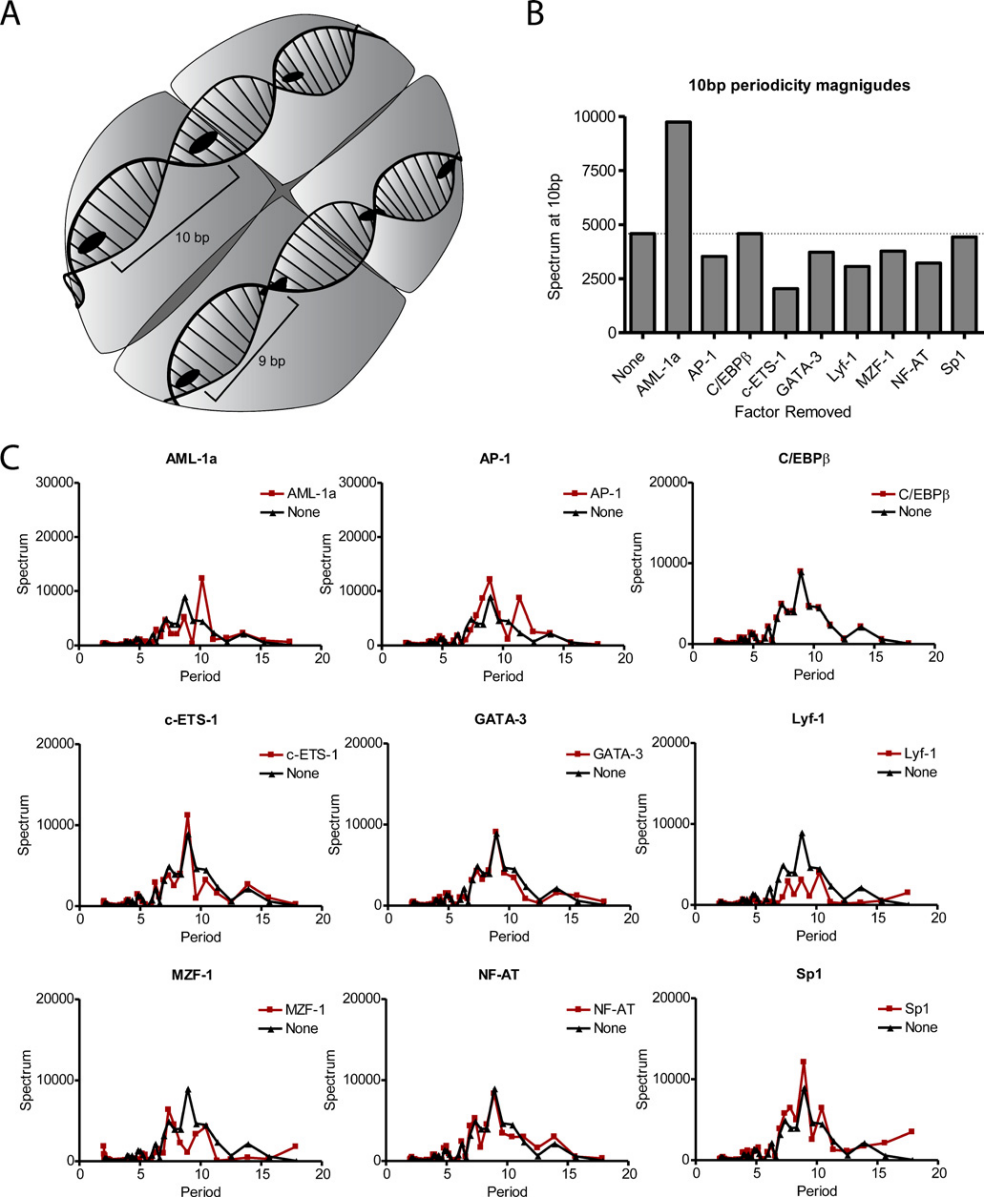
AML-1a specifically lacks 10 bp periodicity within the nucleosome-bound Ly49 regions. (A) Schematic showing the significance of 10 bp periodicity within a nucleosome-bound stretch of DNA. Those factor binding sites that display 10 bp periodicity can be oriented in such a way as to always face out from the nucleosome surface and so still be accessible by transcription factors. Conversely, those factors without 10 bp periodicity are out of phase with the turn of the DNA helix, and so cannot adopt an outward-facing conformation. (B) 10 bp periodicity was determined by Fourier transform of the positional data for all 17 transcription factors pooled together across all nucleosome-bound regions of the Ly49 gene family (first column: ‘None’), or by removing one factor at a time and comparing how this removal affects the periodgram at 10 bp. Note that, because this is a leave-one-out analysis, an increase of 10 bp periodicity upon removing a factor indicates that the removed factor lacks 10 bp periodicity. (C) Individual periodgrams of all transcription factors indicated in (B).

Above, we identified five transcription factor binding sites which are predicted to be covered by nucleosomes in the Ly49 gene family; these transcription factors are the ones we hypothesize to be most sensitive to nucleosome interference. However, as mentioned previously, factors which display a strong 10 bp periodicity are able to exist in-phase with the nucleosome—and therefore be accessible even when covered—while those specifically lacking 10 bp periodicity will be disrupted by the turning of the DNA in the nucleosome (Fig 3A). To measure this effect in the Ly49 gene family, we analyzed the periodicity of each transcription factor, taking an unbiased approach using a leave-one-out analysis. In this analysis, the periodicity of all 17 factors pooled together is determined by Fourier transformation, and then each factor individually is removed from the other 16 to measure the effect its removal has on the overall periodicity. Factors displaying 10 bp periodicity will reduce the overall 10 bp periodicity when removed from the period histogram, while factors without 10 bp periodicity will increase the overall 10 bp periodicity when removed (Figs 3B and 3C and S4B). Based on these results, AML-1a is the only transcription factor that specifically lacks 10 bp periodicity within the Ly49 gene family, as its removal from the pooled factors dramatically increases the signal at 10 bp. Taken together, these results indicate that, of the factors analyzed, AML-1a is the one most sensitive to the surrounding nucleosome environment, as it is the only factor that displays both a tendency to nucleosome coverage (Fig 2) and a specific lack of 10 bp periodicity (Fig 3).

To determine whether AML-1a’s pattern of nucleosome co-occupancy and a lack of 10 bp periodicity is unique to the Ly49 family, we extended these two analyses to the entirety of mouse chromosome 6 (S5 and S6 Figs). To our surprise, while AML-1a retained its nucleosome preference and displayed a very marginal lack of 10 bp periodicity, many of the other transcription factors analyzed displayed much more pronounced coverage (S5 Fig) and an extreme loss of 10 bp periodicity (S6 Fig). This suggests that what we report with regard to AML-1a in Ly49 is one example of a more general paradigm of gene regulation via nucleosomal interference with one or several key transcription factor binding sites.

### Ly49 expression state and nucleosome maps *in vitro* correlate with the predictions

**Fig 4:**
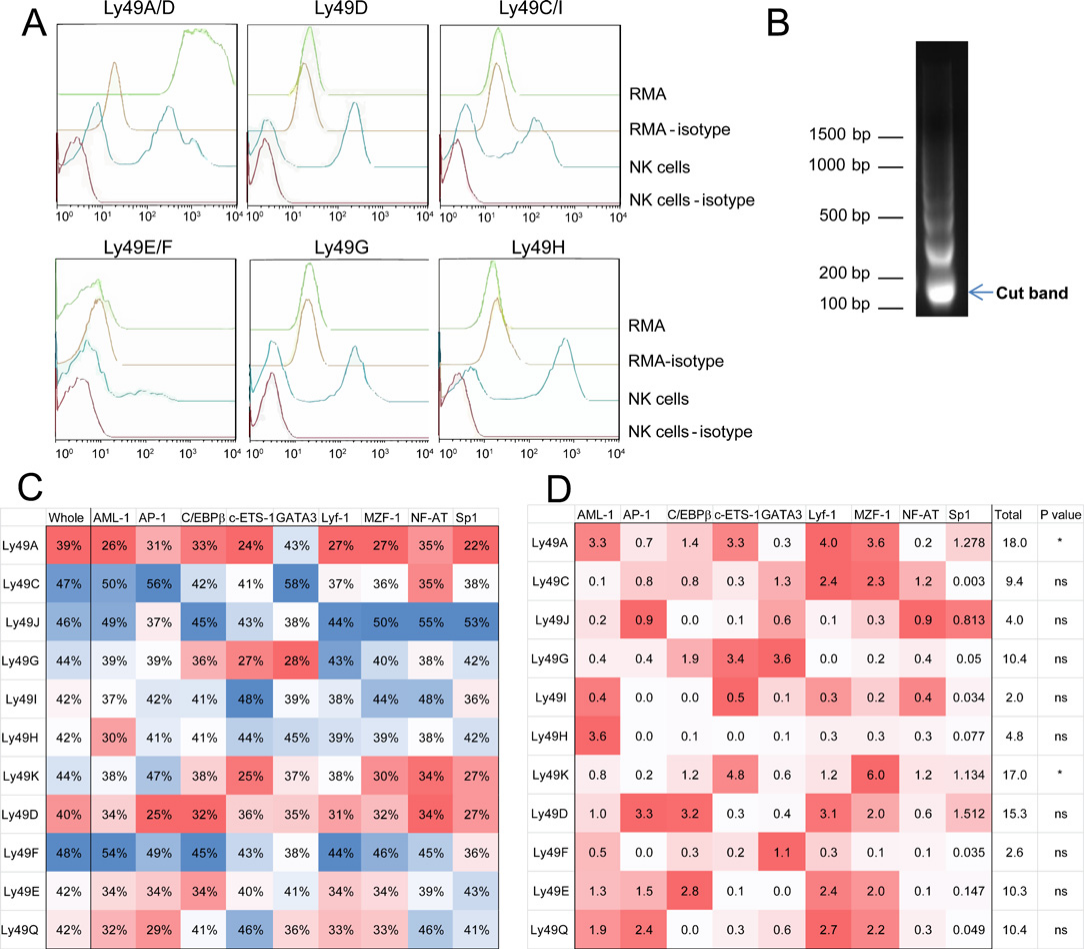
AML-1a shows specific nucleosome depletion *in vitro*. MNase-Seq analysis was performed on the Ly49A-expressing C57BL/6 mouse cell line, RMA, to determine the actual nucleosome positions within the Ly49 gene family. (A) Ly49 expression levels on RMA (green) compared to isotype (yellow), and compared to normal NK cells isolated from C57BL/6 splenocytes as a positive (blue) and negative (isotype, red) control. Note that RMA cells are only positive for Ly49A/D, but are negative for Ly49D, indicating the single expression of Ly49A. (B) Following MNase digest, mononucleosomes were isolated from the ~150 bp fragment and sequenced. (C) Nucleosomes from the MNase-Seq results were compared to the predicted nucleosome landscape to determine the degree of true positive nucleosomes at each Ly49 gene (‘Whole’, first column). This determination was repeated for each of the indicated transcription factors. For each column, a heatmap indicates the relative degree of accuracy of the predictions, with blue cells being more accurate and red being less. Regions with very low accuracy are believed to have had a nucleosome remodeling event. (D) A chi-square analysis was performed to assess the degree of specific nucleosome depletion at the indicated transcription factor binding site, taking the overall nucleosome accuracy (‘Whole’, first column of C) as the expected accuracy for that Ly49. Overall significance of the site-specific nucleosome depletion is indicated, and for each Ly49, the factors most contributing to that significance are indicated in red, with color intensity correlating with contribution to the chi-square statistic. A p value < 0.05 was considered significant.

The Ly49 gene family presents a powerful and convenient tool to test whether these patterns can be detected in an experimental setting. It is a large gene family, whose members all exist in the same region of chromosome 6, and which have closely related promoter sequences and similar transcriptional regulation in terms of transcription factor binding sites. Despite these similarities, individual Ly49 genes can display remarkably different expression patterns. Indeed, RMA, a mouse NK-T cell line with a C57BL/6-derived Ly49 gene cluster, expresses the inhibitory Ly49A receptor, but no other Ly49 for which there is a detecting antibody (Fig 4A). We used this model to determine whether the promoter for Ly49A will have a nucleosome landscape more divergent from the sequence-based predictions than any of the other, inactive Ly49 promoters. We generated the nucleosome sequence map for RMA using MNase-Seq (Fig 4B), and generated a list of nucleosomes that were predicted to be present and confirmed by the MNase-Seq map (‘true positives’) and a list of nucleosomes predicted to be present but found absent in the map (‘false positives’) using the UCSC table browser. Using these lists of true and false positive nucleosome predictions, we generated a heatmap showing the specific accuracy of our predictions for each Ly49 promoter region, across the whole region of interest (‘Whole’) or at all of the sites for each indicated transcription factor (Fig 4C). In this analysis, the promoter region selected includes the areas corresponding to promoters 1, 2, and 3, as well as approximately 8000 bp upstream of Pro1 to capture any distal enhancer elements. This map was coloured to show those Ly49 genes and transcription factor binding sites with a relatively high degree of convergence (blue) or divergence (red) with the prediction.

Next, we asked whether specific TF binding sites, for each promoter individually, diverge significantly in nucleosome occupancy, compared to the rest of that promoter. Taking the overall predictive convergence for each Ly49 promoter (the ‘Whole’ column in Fig 4C) as the expected value, we performed a chi-square analysis on the divergence of the TF binding sites’ nucleosome status from this expected result. Only two Ly49 promoters were significantly divergent in nucleosome occupancy at the tested TF binding sites: the pseudogene Ly49K, and the expressed Ly49A (Fig 4D). All other Ly49 genes are not expressed in RMA, and were not found to significantly differ at TF binding sites from the rest of their nucleosome landscape. As indicated by the heatmap, AML-1a, c-ETS-1, Lyf-1, and MZF-1 were the factors most responsible for the significant divergence in Ly49A (Fig 4D).

**Fig 5:**
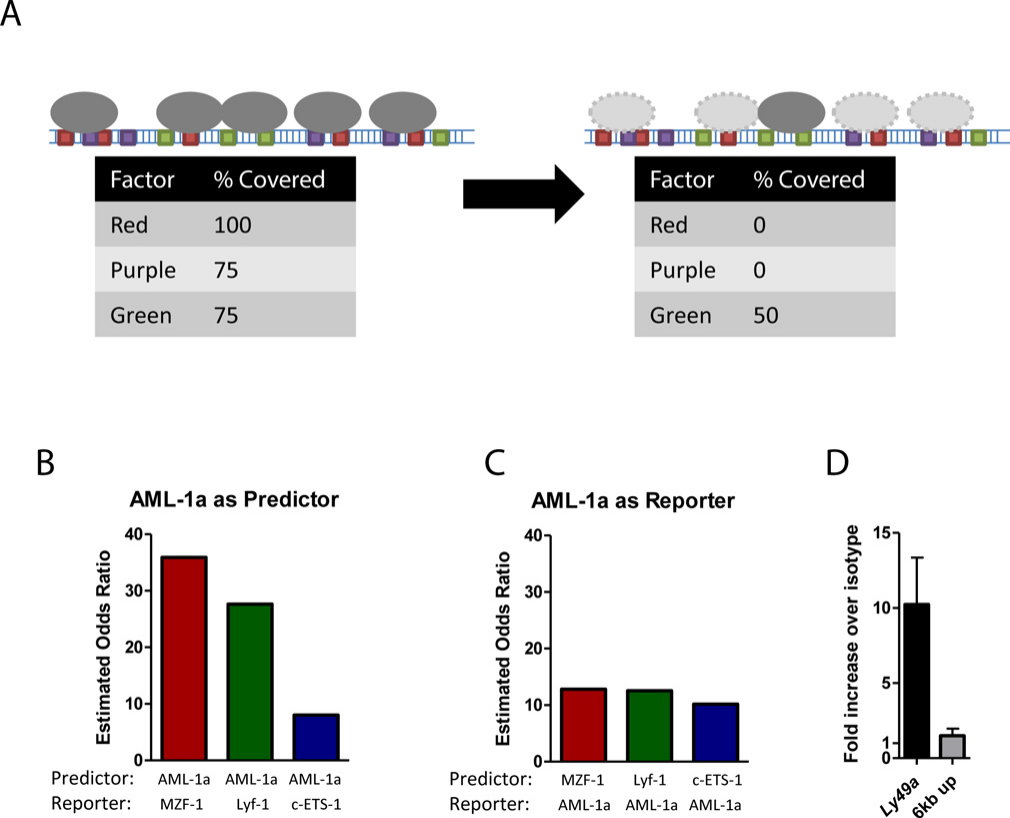
Confounding variables unlikely to account for AML-1a-specific nucleosome depletion in Ly49A. (A) Schematic highlighting the possibility of confounding variables impacting transcription factor/nucleosome relationship studies. If an event occurs to deplete all nucleosomes covering a red factor, the coverage of the red factor drops from 100% to 0%. However, the coverage of all purple factors drops from 75% to 0%, and green from 75% to 50%. Taking nucleosome coverage as the readout, it is impossible to determine whether the red, purple, or green factors ‘caused’ the nucleosome depletion. However, if red depletions are always correlated with purple depletions, while purple depletions only occasionally predict red depletions, it is likely that red is causing the nucleosome remodeling. (B-C) Logistic regression analysis was performed for each of the four most depleted transcription factors from 4C (AML-1a, c-ETS-1, Lyf-1, and MZF-1), using (B) AML-1a as the predictor, or (C) AML-1a as the reporter. In each case, AML-1a was a better predictor of the other two nucleosome-preferring factors, Lyf-1 and MZF-1, than either were of AML-1a. AML-1a performed equally well as predictor or reporter against c-ETS-1, a factor that had no preference for nucleosome coverage, and so was unlikely to be involved as a confounding variable. (D) Chromatin immunoprecipitation (ChIP) using anti-AML-1 or an irrelevant isotype control was performed on RMA chromatin extract. ChIP using the anti-AML-1 antibody resulted in about a 10-fold enrichment of the Ly49A promoter region when compared to ChIP using the isotype control, indicating that AML-1 is likely bound to the promoter. As a control, a region of DNA approximately 6 kbp upstream of the Ly49A promoter displayed no such enrichment.

With the exception of c-ETS-1, these divergent factors are all preferentially covered by nucleosomes (Fig 2), and so exist in close proximity to each other. It is therefore possible that nucleosome divergence at one factor was artificially driving divergence at the others (Fig 5A). To determine whether this could be the case, we performed logistic regression analysis on each pair among these four factors, using nucleosome state (true or false positive) of the predictor to predict the nucleosome state of the nearest binding site for the reporter variable, after controlling for distance between the two sites (specifically, whether the two sites were within 147 bp of each other or not). Knowledge of the state of an AML-1a site was not found to be any better or worse at predicting the state of the nearest c-ETS-1 site than knowledge of a c-ETS-1 site could predict the nearest AML-1a, suggesting that neither of these sites impact the other unequally (Fig 5B and 5C). However, knowledge of the state of an AML-1a site was a much stronger predictor of the state of the nearest MZF-1 or Lyf-1 site than either of these sites was for predicting AML-1a, suggesting that AML-1a-based divergence may be preferentially influencing the Lyf-1- and MZF-1-based divergence (Fig 5B and 5C). Significantly, c-ETS-1 was not one of the factors enriched in nucleosome-bound regions, while AML-1a, Lyf-1, and MZF-1 were preferentially covered factors.

While AML-1 is well-known as a required factor for Ly49 expression in mouse NK cells [35], we performed a chromatin immunoprecipitation to validate the presence of AML-1 at the Ly49A promoter in RMA cells (Fig 5D). Precipitation with anti-AML-1 resulted in a 10-fold enrichment of the Ly49A promoter versus precipitation with an isotype, indicating the presence of AML-1 at this site. Conversely, a genomic region 6 kbp upstream of the Ly49A promoter was not enriched following anti-AML-1 enrichment.

Finally, we analyzed the regions of each Ly49 gene immediately upstream of exon 1 (corresponding to Pro2) and upstream of exon 2 (corresponding to Pro3). As these are much smaller genetic elements with only a few nucleosomes and AML-1a binding sites, statistical analysis similar to the one performed above was not possible. However, for each of Pro2 and Pro3, we identified each AML-1a binding site and whether it was preferentially covered or not, again determining the degree of convergence at these sites (Table 1). Surprisingly, Ly49A displays a higher degree of convergence at AML-1a sites in Pro2 and Pro3 than many of the other, non-expressed Ly49 genes, indicating that the nucleosome divergence for AML-1a in expressed genes is restricted to Pro1 and the enhancer elements.

**Table 1:** Convergence of actual and predicted nucleosomes at the indicated promoter.

Gene	Pro2	Pro3
Ly49A	80%	29%
Ly49C	67%	57%
Ly49J	100%	0%
Ly49G	20%	33%
Ly49I	0%	43%
Ly49H	33%	43%
Ly49D	22%	33%
Ly49F	100%	50%
Ly49E	50%	13%
Ly49Q	100%	20%

## Discussion

In this report, we have shown that nucleosome positioning may be involved in regulating Ly49 expression, not just by condensing the gene complex, but by specifically interfering with AML-1a transcription factor binding sites. This conclusion is based on two complementary studies. *In silico*, we have shown that, within the Ly49 gene cluster, AML-1a is predicted to be exquisitely sensitive to steric hindrance by nucleosome occupancy. The AML-1a binding sites tend to be located within a nucleosome boundary, meaning that AML-1a cannot simply avoid nucleosomes. Moreover, unlike other covered TF binding sites, AML-1a binding sites lack 10 bp periodicity, meaning it must exist ‘out-of-phase’ with the nucleosomes and so cannot adopt a conformation where many AML-1a sites are exposed on the surface of the nucleosome. These predictions were confirmed in a cell line, where we have shown that expressed Ly49 genes show preferential nucleosome depletion specifically at AML-1a binding sites compared to the inactive Ly49 genes. Furthermore, this depletion at AML-1a sites is not likely to be the result of depletion at some confounding TF binding site, since knowledge of the nucleosome depletion of AML-1a was as good or better at predicting the depletion of all other highly depleted TF binding sites than those sites were at predicting AML-1a. Taken together, these results suggest that Ly49 expression is partially regulated by nucleosome occupancy at AML-1a binding sites.

We chose the Ly49 gene family for this analysis for both immunological and bioinformational reasons. Immunologically, Ly49 gene expression on NK cells is an incredibly complex phenomenon, with its mostly stochastic gene expression giving rise to myriad functionally distinct subpopulations of NK cells. This heterogeneity allows a population of NK cells to have members able to respond to virtually any alteration to homeostasis, giving rise to phenomena like missing-self rejection of foreign or diseased cells [41]. As this complex expression of Ly49 receptors is central to NK function, understanding Ly49 transcriptional regulation is central to understanding the NK cell itself, and while studies have focused on *cis*-elements and transcription factors involved in Ly49 regulation, there has been little attention to date given to the role of nucleosome positioning.

From a bioinformatics viewpoint, the Ly49 family presents an attractive subject of study thanks to its genetic isolation, homology, and independent expression. This provides a model system where comparisons can be made between related expressed and silent genes, using one group to control for the other, while any large-scale genetic factors, such as the action of locus control regions or chromosome looping, are likely to impact the entire family equally, regardless of expression state. Additionally, we have taken advantage of this fact to compare expressed and silent Ly49 genes within the same dataset, effectively mitigating the impact of technical errors on our results. We suggest that other predictive informatics endeavours would benefit from performing their analyses on the Ly49 (or KIR, in human datasets) gene family.

Unfortunately, this complex expression of Ly49 does also introduce hindrances into the present study. Not only are the Ly49 genes expressed randomly in a pool of NK cells, but most of them—the inhibitory receptors—display a mono-allelic expression pattern [42,43]. Therefore, any analysis of Ly49 genes from primary NK cells first requires enrichment for cells expressing the Ly49 in question, and then must contend with a sample in which only half of the genomic material includes an Ly49-expressing component. For this reason, we are fortunate that the RMA cell line naturally expresses Ly49A on 100% of the population, as this presents a convenient model for testing our predictions in a natural setting. Future work may focus on performing this analysis across a heterogeneous pool of natural killer cells *ex vivo*, which among other things would allow for the study of the differences between inhibitory and activating Ly49 receptors. Unfortunately, such a complex study goes beyond the scope of the present report.

That our analysis identifies and confirms AML-1a binding sites—specifically within the Pro1/enhancer region—as the most significant in terms of nucleosomal regulation is intriguing, thanks to AML-1’s known role in Ly49 transcriptional control. One of the features of the Pro1 region in many Ly49 genes is a central AML-1a binding site, identified as being absolutely required for Pro1 to function in either direction [35]. Thus, disruption of this AML-1a binding site represents a potent mechanism of Ly49 expression suppression, and may explain why nucleosomes appear to target AML-1a binding sites within unexpressed Ly49 genes. Interestingly, the requirement of the AML-1a site for both forward and reverse transcription at this site suggests that nucleosome regulation is unlikely to directly impact the stochastic expression of an Ly49 gene, but rather interferes with any gene activity at all, either forward or reverse. Whether this loss of nucleosome occupancy in expressed Ly49 genes is a cause or an effect of Ly49 gene transcription remains to be determined. That the nucleosome regulation is found within the Pro1/enhancer region may also indicate that nucleosomes best exert their regulatory effects during cell development and maturation, as Pro1 is active only in developing NK cells.

Finally, it will be of interest to study the nucleosome sensitivity of AML-1a binding sites at other, non-Ly49 genes, to determine whether AML-1a is ubiquitously sensitive to nucleosome occupancy, or whether this sensitivity is a selected trait for Ly49 regulation only. Our whole-chromosome analysis of nucleosome-transcription factor interactions revealed the surprising result that, on this scale, many other transcription factors display the nucleosome coverage and lack of 10 bp periodicity that identified AML-1a as a sensitive factor in the Ly49 cluster. This suggests that, on a genome-wide scale, many different transcription factors have the potential to be nucleosome-sensitive. However, within a given gene or gene family, only a small number of factors—such as AML-1a in Ly49—retain this sensitivity, and may act as nucleosome ‘linchpin’ factors, while the other factors lose their sensitivity either by avoiding nucleosomes or by getting in-phase with them. A genome-wide characterization and identification of these linchpin factors and their associated genes could provide a novel, informative method of functionally grouping genes.

It is our hope that this analysis of TF distribution and periodicity will assist in achieving a greater understanding of how chromatin compaction and nucleosome positioning shapes the gene expression landscape.

## Materials and Methods

### Prediction of nucleosome binding sites

The genomic DNA sequence of the Ly49 gene cluster from C57BL/6 mice was previously available [44]; sequences for 129, BALB/c, and NOD mice were generated previously by A.P.M. [45–47]. These genomic DNA sequences were used to predict the nucleosome affinity and the probability of nucleosome binding by NuPoP [48]. The prediction parameter was set as the 4th order-Hidden Markov Model, and the species was set as mouse. NuPoP produces nucleosome occupancy score, affinity score, and Viterbi prediction of the position. The nucleosome dyad was set as the middle of the nucleosome position determined by the Viterbi prediction.

### Prediction of transcription factor binding sites

The same four genomic DNA sequences of the Ly49 gene clusters of the four mouse strains were used to predict transcription factor binding sites. The Position Weight Matrix (PWM) of the 18 transcription factors (AML-1a, AP-1, C/EBPβ, Egr-1, Egr-2, Egr-3, GATA-3, IRF-1, Ik-3, Lyf-1, MZF1, NF-AT, NF-κB, Oct-1, STAT3, Sp1, Tal-1α/E47, c-Ets-1(p54)) were retrieved from the TRANSFAC or JASPAR public databases [49,50]. Detection of predicted TF binding sites was performed using either the MATCH or FIMO algorithm [51,52], with cut-off selection set to minimize false positives. The TATA binding site position was also predicted as a control.

### Nucleosome landscape around Pro1 region

The promoter architecture was presented around the Pro 1 region including Pro1 and exon -1a. The landscape of the nucleosome binding sites and the TF binding sites in the promoters were examined to find relationships. Nucleosome predictions were aligned to exon -1a for each Ly49 gene in the C57BL/6 mouse. For genes without an exon -1a annotation, the average distance between the Pro1 and exon -1a for each annotated gene was used to approximate the location of exon -1a. If a transcription factor binding site was overlapped with a nucleosome position, then the transcription factor binding site was marked as closed. Otherwise, it was marked as open.

### Distribution of transcription factor binding sites around the nearest nucleosome position

The distribution of transcription factor binding sites around the proximal nucleosome was explored for each transcription factor. First, the output files of the transcription factor binding sites and the Viterbi prediction of the nucleosome positions were converted to BED format. Then for each transcription factor binding site, the proximal nucleosome was identified by the BEDtools suite [53] using the CLOSEST command. The distance was calculated between the middle position of the transcription factor binding site and the dyad position of the proximal nucleosome. The distances from all pairs of the transcription factor binding site and the proximal nucleosome were counted to generate the histogram. The density of the distribution was estimated using Kernel Density Estimation by R software. The distribution was centered at the nucleosome dyad, with boundaries marked at 73 bp away from the dyad on either side.

### Computation of 10 bp periodicity for each transcription factor using a leave-one-out test

For all transcription factor binding sites located within the predicted nucleosome-bound regions, we examined the periodicity of the distances between the transcription factor binding sites and the proximal nucleosome by Fourier transform. For each nucleosome-bound region of the Ly49 cluster, the distance between each transcription factor and its proximal nucleosome center was selected and pooled. The spectral density of the pooled distance counts from the 17 transcription factors was generated as a baseline diagram. Then, the spectral density of the distance counts from leave-one-out samples, which are the counts from 16 transcription factors by excluding one transcription factor at a time, were computed. The periodicities from the leave-one-out samples were compared with the baseline periodicities. Deviation from the baseline indicated that the left-out factor contributed to the periodicity of the baseline—with the factor gone, a reduction in periodicity at the 10 bp peak indicates that the left-out factor contributed to the overall 10 bp periodicity.

### Nucleosome map of RMA cells

The predicted binding positions were compared with the experimental positions by MNase-seq results obtained on the mouse NK-T cell line, RMA, as previously described [54], with the following changes: library preparation was performed by Génome Quebec at McGill University, and samples were prepared and analyzed on an Illumina MiSeq (Illumina, San Diego, CA). MNase-seq results have been deposited in the GEO database, under accession GSE71863. The intersections of predictions with nucleosome-bound-regions (true predictions) or nucleosome-free regions (false predictions) were found using the UCSC table browser. The same approach was repeatedly used for each transcription factor binding site to identify the number of sites correctly or incorrectly predicted to be covered by a nucleosome. The degree of accuracy of the prediction was calculated for nucleosomes in general and for each TF binding site, performed for each Ly49 gene. The results are presented as a heatmap between the nucleosome predictions and the transcription factors.

A chi-square analysis was performed to detect specific transcription factors enriched for nucleosome deviancy compared to the rest of the gene. Based on each gene’s overall true-positive rate, expected true-positive counts were generated for each Ly49 region of interest. These expected values were compared to the observed using the chi-square test. To highlight individual TF contributions to the region of interest’s overall chi-square statistic, individual chi-square statistics were presented in a heat-map.

Odds ratios were estimated by logistic regression for each of the indicated TFs and AML-1a. Regression was performed using R by comparing the state (true or false positive coverage) at each predictor site to the nearest reporter site in the Ly49 cluster, after accounting for whether the two sites were within 147bp or not. Higher estimated odds ratios indicate that knowledge of the state of the predictor site is more able to predict the state of the reporter site.

### Ly49A expression on RMA cells

RMA cells or isolated mouse splenocytes were analyzed by flow cytometry after staining with antibodies against NK1.1, TCRβ, and the Ly49 receptors such as Ly49A/D (clone 4E5), Ly49D, Ly49C/I (5E6), Ly49E/F, Ly49G (4D11), and Ly49H (3D10). Antibodies were purchased from eBioscience (San Diego, CA) or Becton Dickinson (San Jose, CA), and samples were acquired using a Beckman Coulter CyAN-ADP and analyzed using FlowJo (FlowJo LLC, Ashland, OR).

### Chromatin immunoprecipitation of RMA cells

Fragmented chromatin from RMA cells was prepared by MNase digest as above, except that 150 bp fragments were not selected. Chromatin was then incubated with a rabbit polyclonal antibody raised against AML-1 or against immunoglobulin M (as an isotype control) overnight (Abcam). Antibody complexes were collected using protein A agarose beads, and DNA was purified using a high pH chelating solution, as previously described [55,56]. The following primers were used:

Ly49A promoter: AGGCCAGGGAAACCTGGTGTA

AAGAGGTGGGGCACTGGACTG

Ly49A 6 kb up: ACAGAACTCAGAGGGCAAAGGAAA

TGGGCCACTTGGCCATTTATCT

Real-time PCR was performed using an Eppendorf Realplex^2^ Mastercycler thermal cycler.

## Supporting Information

S1 Fig Hidden Markov prediction of nucleosome binding sites. (A) Example of an alignment showing the raw genomic affinity (1^st^ track) and probable occupancy (2^nd^ track) of the Ly49G pro1 region (3^rd^ track). Transcription factor binding sites from the TransFac database (4^th^ track) are also shown. (B) Analysis of %GC bias introduced by nucleosome prediction. (C) Prediction validation by comparison to a published MNase-Seq dataset. Nucleosome predictions (in black) were tested against a published MNase-Seq dataset from the Gene Expression Ontology project (GEO, accession number GEO58005), presented in blue. Nucleosomes having at least 50% overlap with the prediction were taken to represent a true prediction (green), while other nucleosomes were taken as false (red). An example window is shown, with a total accuracy of 48.8% correctly identified nucleosomes.
(TIF)

S2 Fig Predicted nucleosome hindrance in other mouse genomes. Probable nucleosome position across the Ly49 gene family is shown as in Fig 1 for three different mouse strains: (A) 129S6, (B) BALB/c, (C) and NOD mice. Nucleosome-bound and nucleosome-free regions are indicated as before, again showing a trend toward a free reverse promoter 1 and a bound forward promoter 1.
(TIF)

S3 Fig Transcription factor binding sites used in this study. Position weight matrices obtained from the TRANSFAC or JASPAR public databases are shown as sequence logos. Logos were generated using the seqLogo R package from Bioconductor.
(TIF)

S4 Fig Nucleosome proximity and nucleosome-bound periodicity of rare transcription factor binding sites. Of the 17 transcription factors studied in this report, 8 were excluded due to insufficient binding sites (< 300) in the Ly49 gene family. The (A) nucleosome proximity and (B) nucleosome-bound periodicity for these excluded factors are indicated here.
(TIF)

S5 Fig Nucleosome proximity of all factors across mouse chromosome 6. Factor-nucleosome distances were calculated and presented as in Fig 2. As the whole chromosome was used, all factors gave sufficient signal to generate histograms.
(TIF)

S6 Fig Transcription factor periodicity for all predicted nucleosome-bound regions in chromosome 6. Factor periodicities were calculated and presented as in Fig 3. Graphs show the periodgram of the entire amalgam (black) and the effect of removing the indicated factor from the amalgam (red). The histogram highlights the effect of removing each factor individual on the 10 bp periodicity.
(TIF)
