# Supplementary figures and images for: Nucleosome Presence at AML-1 Binding Sites Inversely Correlates with Ly49 Expression: Revelations from an Informatics Analysis of Nucleosomes and Immune Cell Transcription Factors
Source: PLoS Comput Biol. 2016 Apr 28;12(4):e1004894. doi: 10.1371/journal.pcbi.1004894 (PMC4849748; doi:10.1371/journal.pcbi.1004894)

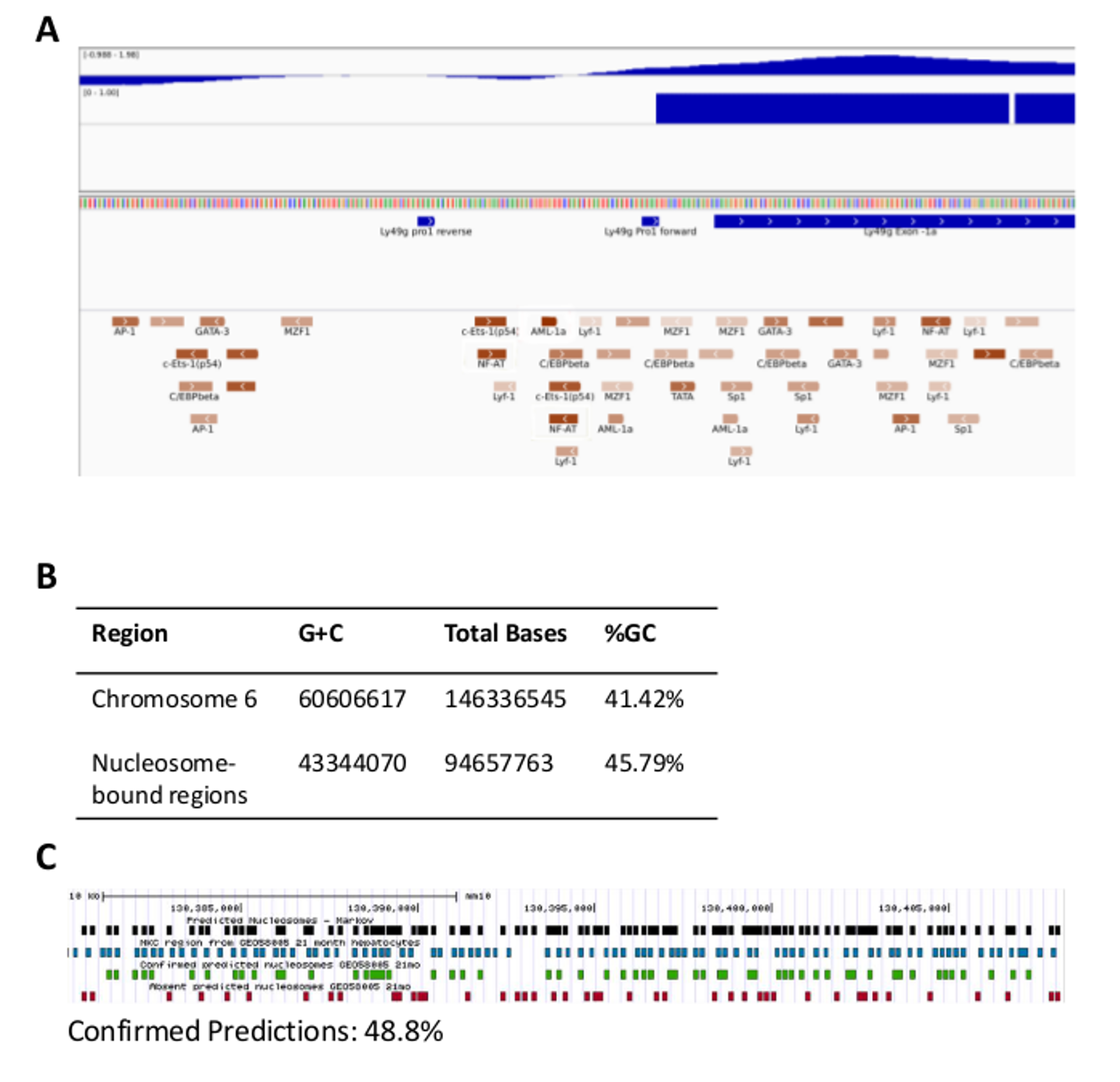

Supplement: S1 Fig — (A) Example of an alignment showing the raw genomic affinity (1st track) and probable occupancy (2nd track) of the Ly49G pro1 region (3rd track). Transcription factor binding sites from the TransFac database (4th track) are also shown. (B) Analysis of %GC bias introduced by nucleosome prediction. (C) Prediction validation by comparison to a published MNase-Seq dataset. Nucleosome predictions (in black) were tested against a published MNase-Seq dataset from the Gene Expression Ontology project (GEO, accession number GEO58005), presented in blue. Nucleosomes having at least 50% overlap with the prediction were taken to represent a true prediction (green), while other nucleosomes were taken as false (red). An example window is shown, with a total accuracy of 48.8% correctly identified nucleosomes. (TIF) [file pcbi.1004894.s001.tif]

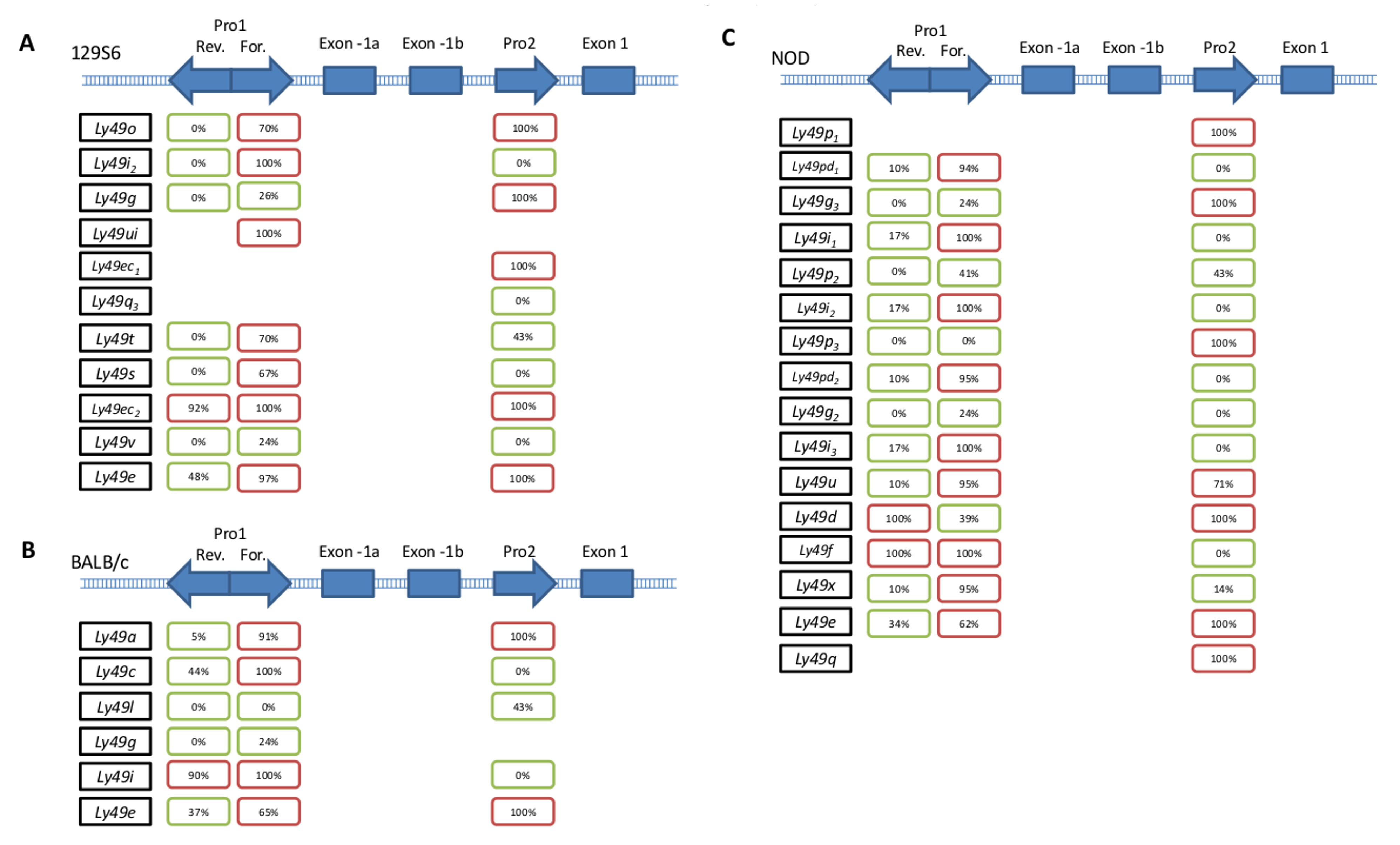

Supplement: S2 Fig — Probable nucleosome position across the Ly49 gene family is shown as in Fig 1 for three different mouse strains: (A) 129S6, (B) BALB/c, (C) and NOD mice. Nucleosome-bound and nucleosome-free regions are indicated as before, again showing a trend toward a free reverse promoter 1 and a bound forward promoter 1. (TIF) [file pcbi.1004894.s002.tif]

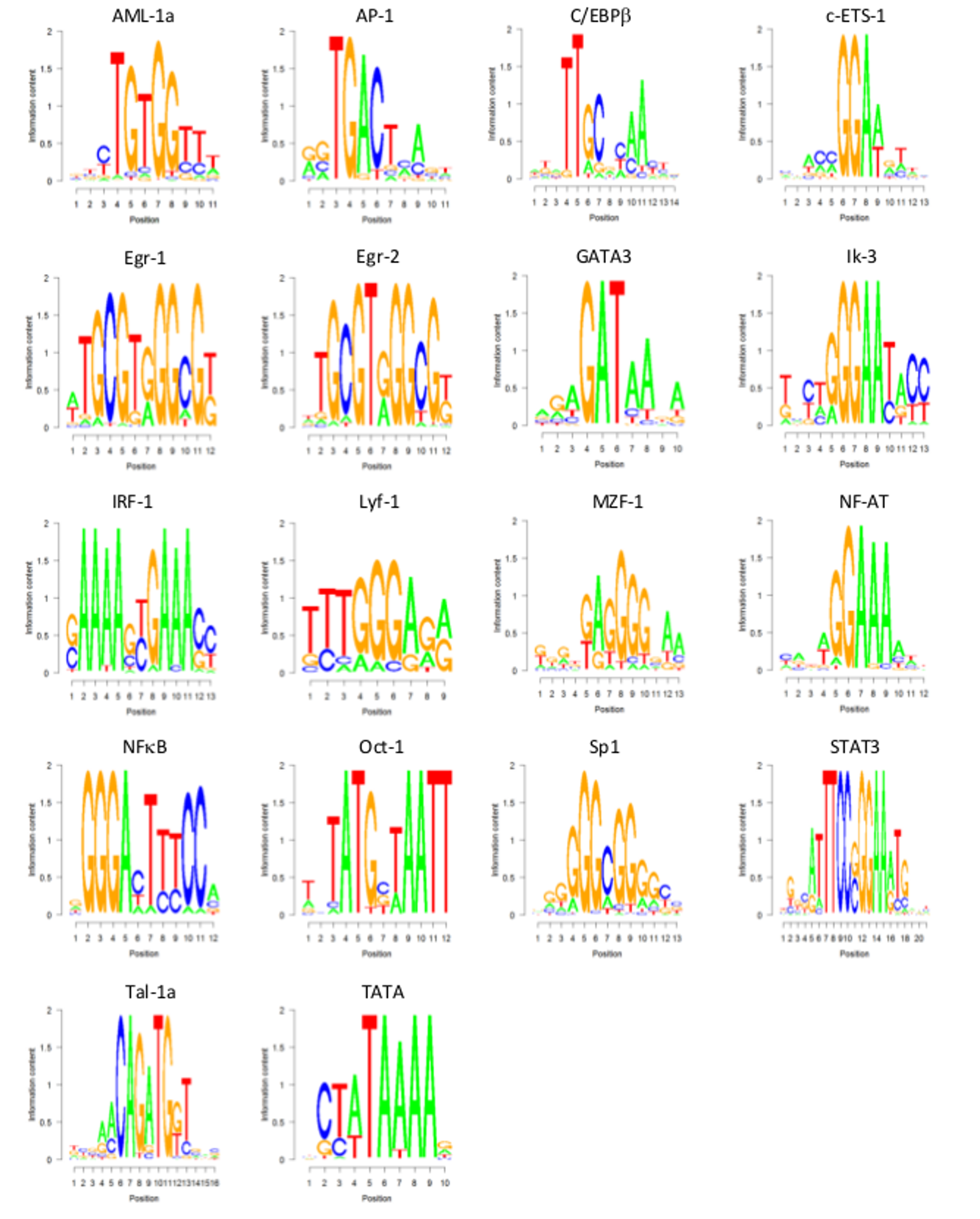

Supplement: S3 Fig — Position weight matrices obtained from the TRANSFAC or JASPAR public databases are shown as sequence logos. Logos were generated using the seqLogo R package from Bioconductor. (TIF) [file pcbi.1004894.s003.tif]

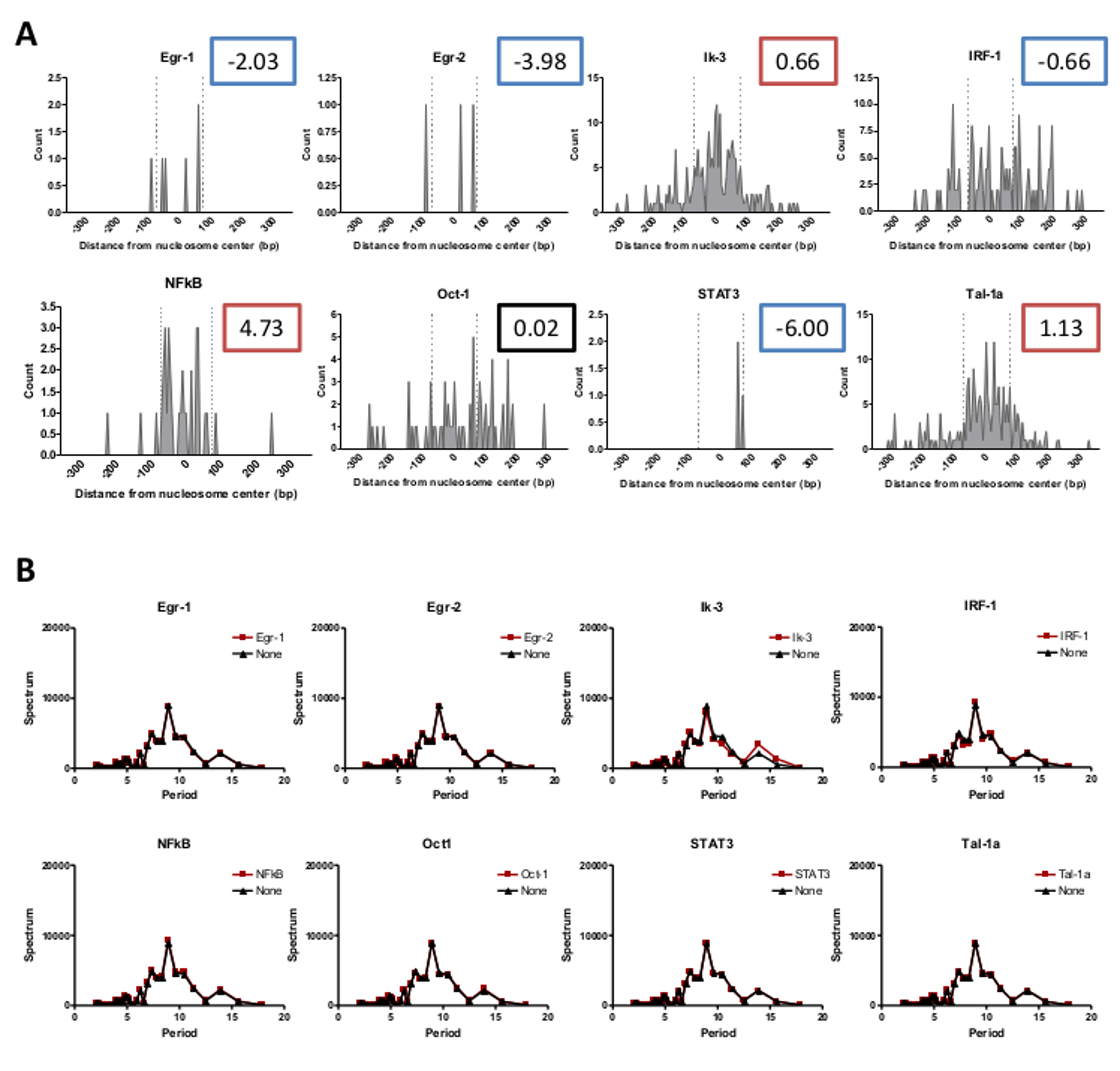

Supplement: S4 Fig — Of the 17 transcription factors studied in this report, 8 were excluded due to insufficient binding sites (< 300) in the Ly49 gene family. The (A) nucleosome proximity and (B) nucleosome-bound periodicity for these excluded factors are indicated here. (TIF) [file pcbi.1004894.s004.tif]

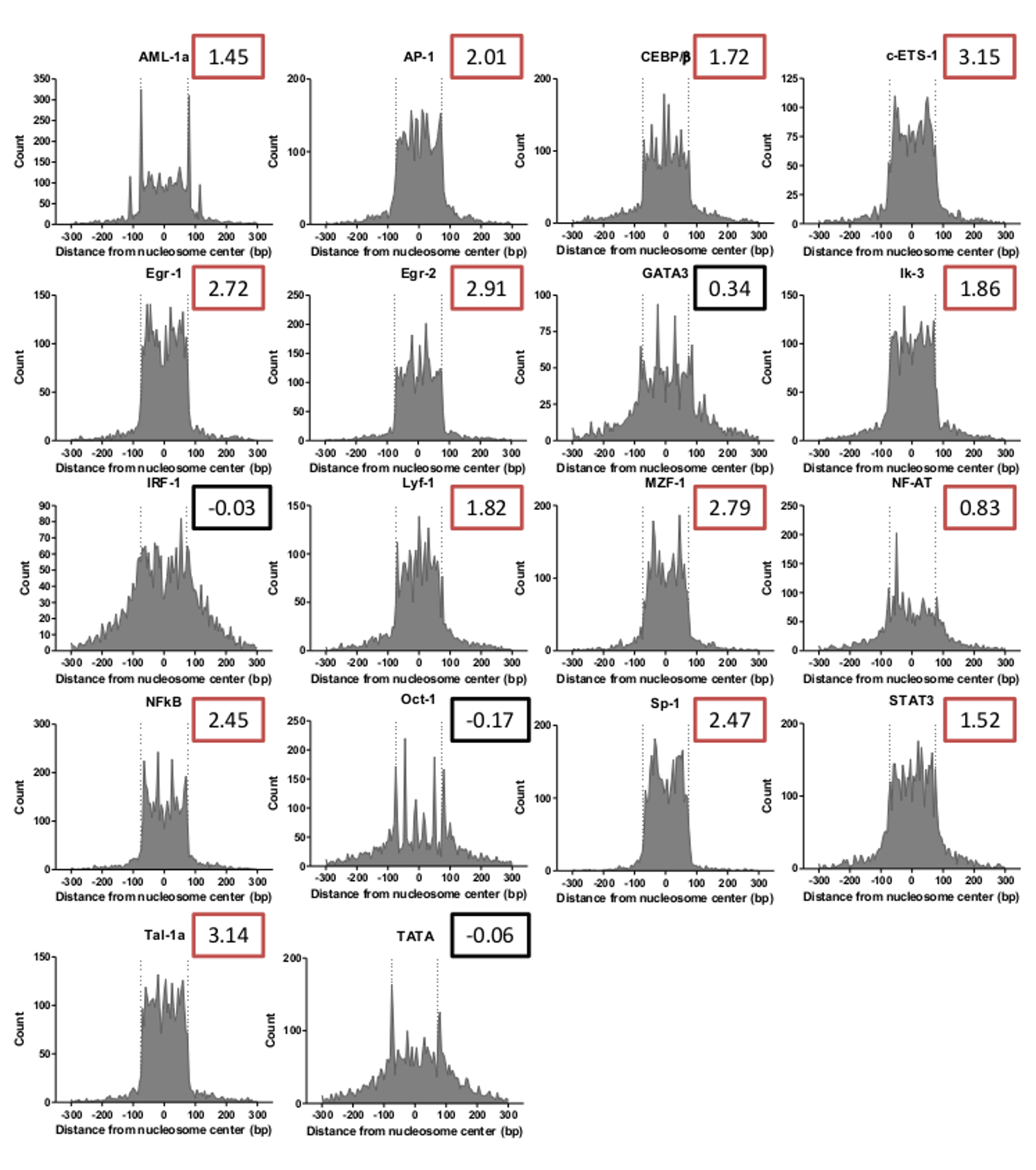

Supplement: S5 Fig — Factor-nucleosome distances were calculated and presented as in Fig 2. As the whole chromosome was used, all factors gave sufficient signal to generate histograms. (TIF) [file pcbi.1004894.s005.tif]

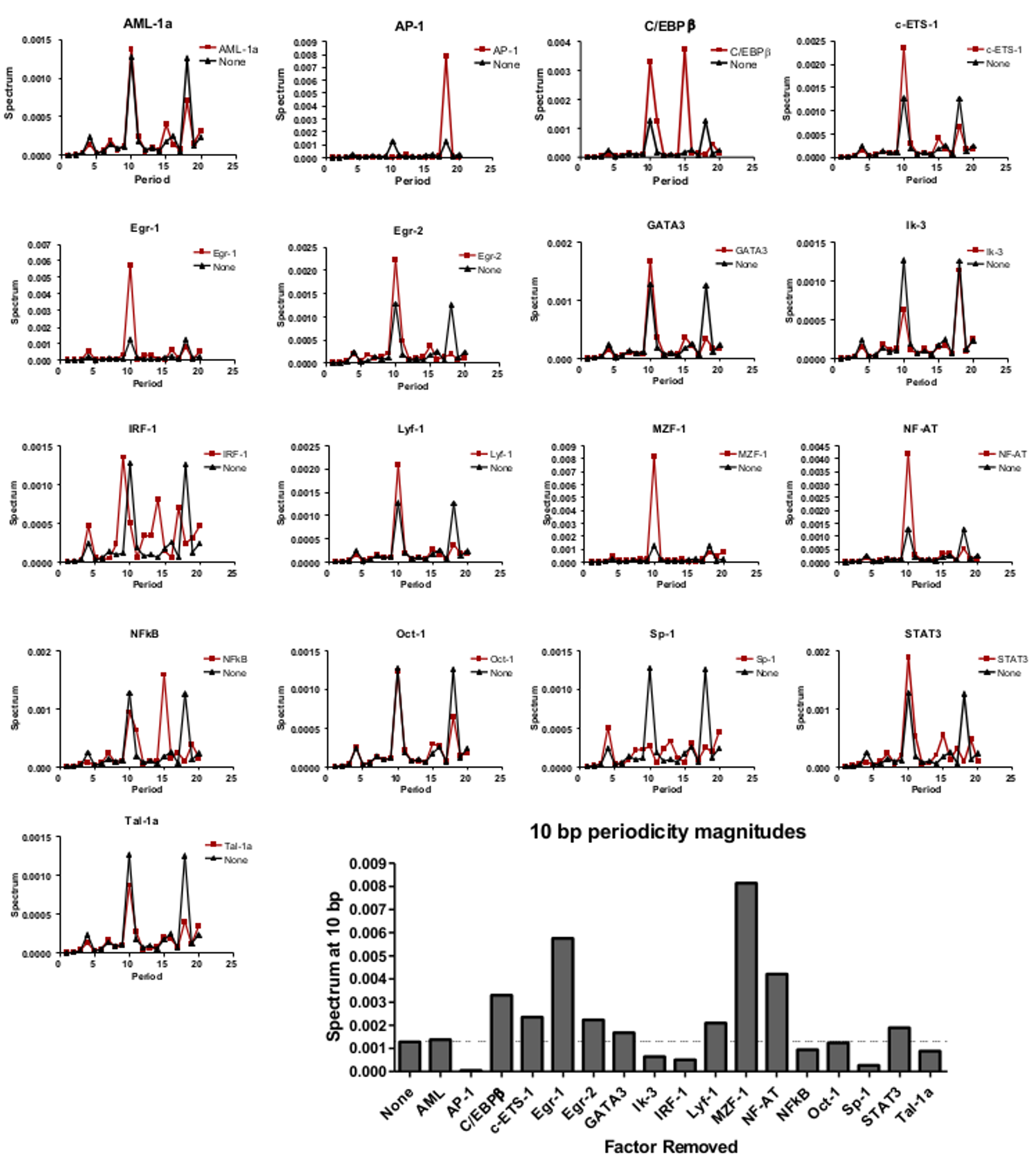

Supplement: S6 Fig — Factor periodicities were calculated and presented as in Fig 3. Graphs show the periodgram of the entire amalgam (black) and the effect of removing the indicated factor from the amalgam (red). The histogram highlights the effect of removing each factor individual on the 10 bp periodicity. (TIF) [file pcbi.1004894.s006.tif]
